# Designing the Optimal Digital Health Intervention for Patients’ Use Before and After Elective Orthopedic Surgery: Qualitative Study

**DOI:** 10.2196/25885

**Published:** 2021-03-08

**Authors:** Anna Robinson, Robert D Slight, Andrew K Husband, Sarah P Slight

**Affiliations:** 1 School of Pharmacy Newcastle University Newcastle upon Tyne United Kingdom; 2 Population Health Sciences Institute Newcastle University Newcastle upon Tyne United Kingdom; 3 Newcastle upon Tyne Hospitals NHS Foundation Trust Newcastle upon Tyne United Kingdom

**Keywords:** digital technology, orthopedic surgery, behavior change, perioperative care, prehabilitation, qualitative research, mHealth, eHealth, mobile phone

## Abstract

**Background:**

Health behavior changes made by patients during the perioperative period can impact the outcomes and success of elective surgeries. However, there remains a limited understanding of how best to support patients during this time, particularly through the use of digital health interventions. Recognizing and understanding the potential unmet needs of elective orthopedic surgery patients is central to motivating healthier behavior change, improving recovery, and optimizing overall surgical success in the short and long term.

**Objective:**

The aim of this study is to explore patient perspectives on technology features that would help support them to change their lifestyle behaviors during the pre- and postoperative periods, and that could potentially maintain long-term healthy lifestyles following recovery.

**Methods:**

Semistructured interviews with pre- and postoperative elective orthopedic patients were conducted between May and June 2020 using telephone and video call–based software. Patient perspectives on the use of digital technologies to complement current surgical care and support with lifestyle behavior changes were discussed. Interviews were audio recorded and transcribed verbatim. Reflexive thematic analysis enabled the development of themes from the data, with QSR NVivo software (version 12) facilitating data management. Ethical approval was obtained from the National Health Service Health Research Authority.

**Results:**

A total of 18 participants were interviewed. Four themes were developed from the data regarding the design and functionality of digital technologies to best support the perioperative journey. These center around an intervention’s ability to incorporate interactive, user-centered features; direct a descriptive and structured recovery; enable customizable, patient-controlled settings; and deliver both general and specific surgical advice in a timely manner. Interventions that are initiated preoperatively and continued postoperatively were perceived as beneficial. Interventions designed with personalized milestones were found to better guide patients through a structured recovery. Individualized tailoring of preparatory and recovery information was desired by patients with previously high levels of physical activity before surgery. The use of personalized progression-based exercises further encouraged physical recovery; game-like rewards and incentives were regarded as motivational for making and sustaining health behavior change. In-built video calling and messaging features offered connectivity with peers and clinicians for supported care delivery.

**Conclusions:**

Specific intervention design and functionality features can provide better, structured support for elective orthopedic patients across the entire surgical journey and beyond. This study provides much-needed evidence relating to the optimal design and timing of digital interventions for elective orthopedic surgical patients. Findings from this study suggest a desire for personalized perioperative care, in turn, supporting patients to make health behavior changes to optimize surgical success. These findings should be used to influence future co-design projects to enable the design and implementation of patient-focused, tailored, and targeted digital health technologies within modern health care settings.

## Introduction

### Background

Digital health technologies are becoming increasingly common in various industries, with medicine, surgery, and health care being no exception [1]. The use of digital health interventions is growing significantly among patients and health care providers, with recent studies reporting over 318,000 smartphone apps available to aid in health education, diagnosis, and self-management [2-4]. Despite the multitude of digital solutions available, many fail to meet patient and provider expectations—with their use and uptake hindered by ethical issues such as privacy and security of data, disease management, and communication [5]. Involving technology end users in cocreation approaches has been acknowledged as a possible strategy to design digital interventions that meet both the patient and provider needs [6,7].

In recent digital health literature, there are various interventions that have successfully supported patients in managing long-term health conditions [8] and medication adherence [9,10] and supporting positive lifestyle behavior change before and after surgery to improve postoperative outcomes [11,12]. Health behavior changes made during the perioperative period can be fundamental in determining the outcomes and success of elective surgeries. In the context of orthopedic surgery, increases in preoperative physical activity levels and smoking cessation have been associated with improved postoperative bone healing [13], wound healing [14], quicker recovery times, and reduced pain scores [15]. Physical rehabilitation after orthopedic surgery is an essential component of treatment, as it helps to improve functional outcomes and support patients to return to their daily activities [16]. There remains a limited understanding of how best to support patients during this time, particularly through the use of digital interventions.

In this context, research has focused on the orthopedic clinician’s use of digital technologies [1,17], for instance, in supporting their educational development [18], guiding clinical decision support [19], managing care referrals [20], and building the patient-clinician relationship [21,22]. Recognizing and understanding the potential unmet needs of elective orthopedic surgery patients is central to motivating healthier behavior change, improving their recovery, and optimizing overall surgical success in the short and long term [23-25]. The optimal design and functionality of digital solutions to aid this cohort are yet to be recognized.

### Objectives

To develop useful and effective digital technologies and strategies, it is important to first understand how patients want to be supported on their care pathway. Our patient-informed research applies qualitative investigation to explore patient perspectives and identify key technology features that they would find supportive during the pre- and postoperative periods and that could potentially maintain long-term healthy lifestyles following recovery. Specifically, our key research questions concerned the following: (1) *What* do orthopedic patients want from digital health technologies? (2) *How* do they want to use them? and (3) *When* would they be of most benefit during their elective surgical journey?

## Methods

### Recruitment and Sampling

The Consolidated Criteria for Reporting Qualitative Research checklist was followed for this study, according to Enhancing the QUAlity and Transparency Of health Research guidelines (Multimedia Appendix 1) [26]. Immediately before study commencement, COVID-19 restrictions were enforced across the United Kingdom. This meant that the planned face-to-face recruitment and data collection could no longer be undertaken in person at one of the largest teaching hospitals in North England. Instead, an amendment to the National Health Service (NHS) Health Research Authority (HRA) Ethics meant that participants could be recruited remotely via email and social media. All participants were emailed with an information sheet detailing the purpose and aim of this study. Participants who expressed an interest and provided written consent were enrolled in the study. There was no prior relationship established between the researcher and participants before study commencement or recruitment. Inclusion criteria were as follows: participants aged more than 18 years who were due to undergo (or had recently undergone, within the last 2 years) elective orthopedic surgery, who were medically stable and did not have an acute decline in health away from their baseline, who were able to participate in an interview, who were able to communicate in English, and who had the capacity to consent to participate in the study. Purposive sampling was used to recruit participants undergoing a variety of orthopedic surgical procedures, with mixed age ranges and sociodemographic backgrounds.

### Semistructured Interviews

In-depth, semistructured interviews were conducted by 1 researcher (AR, a female doctoral researcher with experience in qualitative research) between May and June 2020 while working from home. Interviews were conducted with participants over the telephone or by using video call–based software, such as Zoom and Microsoft Teams, and all participants were offered the choice of which they would prefer. The semistructured interview topic guide was developed based on 3 pilot interviews and covered key issues identified through a systematic literature review [12], meta-ethnography [27], and narrative review [28]. These issues included participants’ understanding and experiences of surgery, awareness of perioperative lifestyle behavior change, perspectives on digital health technology use within the surgical pathways, and the optimal design of such technologies.

### Data Analysis

All interviews were audio recorded and transcribed verbatim by 1 researcher (AR). All data were anonymized at the point of transcription; participants did not comment on the transcripts or provide feedback on results. Following reflexive thematic analysis processes, as defined by Braun and Clarke [29,30], each interview was transcribed and analyzed before conducting the next interview. The principle of constant comparison guided the iterative process of data collection and analysis. Two researchers (AR and AH) performed a reflexive thematic analysis to analyze the data. Close and detailed reading of the transcripts allowed the 2 researchers to familiarize themselves with the data. Initial descriptive codes were identified in a systematic manner across the data set; these were then sorted into common coding patterns, which enabled the development of analytic themes from the data. The themes were reviewed, refined, and named once coherent and distinctive. Two authors (AR and AH) performed the data analysis through discussion and, if agreement was not reached, by consensus with the wider research team (SS and RS). The postinterview field notes enhanced this reflective process. QSR NVivo software (version 12) was used to facilitate data management. The research team agreed that data saturation occurred in 18 interviews. To ensure confidentiality when using direct patient quotes within this research, nonidentifiable pseudonyms are used throughout, for example, participant 1 and participant 2.

### Ethical Approval

Ethical approval was obtained from the NHS HRA and Care Research Wales (reference: 19/NE/0318), and research governance was granted by the participating NHS trust.

## Results

### Overview

A total of 18 participants were recruited and interviewed as part of this study (there were no refusals to partake, participant dropout, or repeat interviews). The characteristics of participants are presented in Table 1. The average age of the participants was 52 (SD 16.7) years, and the most common elective orthopedic procedure was a total hip replacement. A total of 11 interviews were conducted over the telephone and 7 were conducted using video call–based software. The average duration of the interview was 48 (SD 8.5) minutes.

**Figure 1:**
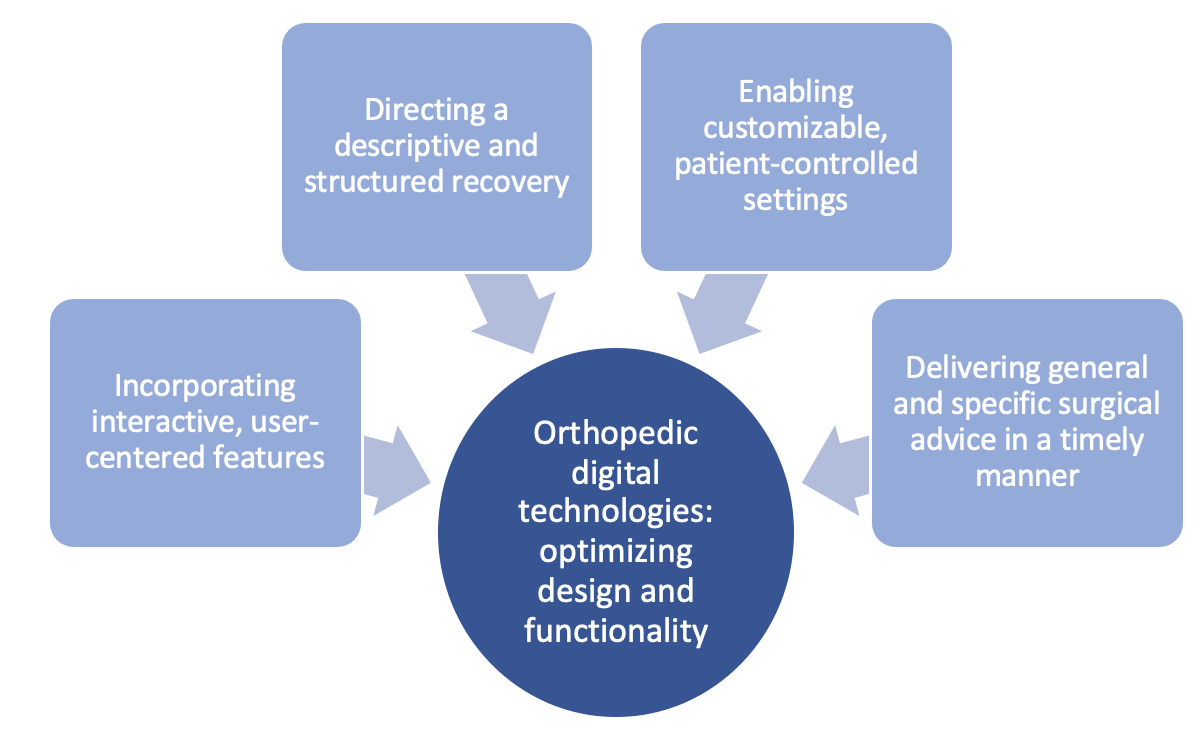
Features contributing to the design and functionality of digital technologies to best support orthopedic patients in their perioperative journey.

Four themes were developed from the data (Figure 1) that addressed the aforementioned research questions. These themes centered on an intervention’s ability to (1) incorporate interactive, user-centered features; (2) direct a descriptive and structured recovery; (3) enable customizable, patient-controlled settings; and (4) deliver both general and specific surgical advice in a timely manner. We will discuss each of these themes, in turn, illustrating patient perspectives and recommendations with direct interview quotes.

**Table 1:** Participant characteristics.

Participant number	Sex (M^a^ or F^b^)	Age (years)	Interview format	Orthopedic procedure	Pre- or postoperative	Time since surgery or time until surgery
1	F	83	Telephone	TKR^c^	Post	12 m^d^
2	M	63	Telephone	TKR	Post	6 m
3	M	63	Telephone	TKR	Post	24 m
4	F	41	Video call	THR^e^	Post	22 m
5	F	42	Video call	THR	Post	14 m
6	M	61	Telephone	THR	Post	20 m
7	M	70	Telephone	THR	Post	16 m
8	F	50	Telephone	THR	Post	8 m
9	F	69	Telephone	THR	Post	24 m
10	M	50	Video call	THR	Post	10 m
11	M	66	Telephone	TKR	Pre	2 w^f^
12	M	26	Video call	Hip FAIS^g^	Pre	4 w
13	F	62	Telephone	WL R^h^	Pre	6 w
14	M	26	Video call	ACL R^i^	Post	6 w
15	F	30	Telephone	Ankle reconstruction	Pre	1 w
16	M	24	Video call	ACL R	Post	6 m
17	M	56	Telephone	TKR	Pre	3 w
18	M	54	Video call	THR	Pre	8 w

^a^M: male.

^b^F: female.

^c^TKR: total knee replacement.

^d^m: months.

^e^THR: total hip replacement.

^f^w: weeks.

^g^FAIS: femoral acetabular impingement surgery.

^h^WL R: wrist ligament reconstruction.

^i^ACL R: anterior cruciate ligament reconstruction.

### Theme 1: Incorporating Interactive, User-Centered Features

When considering *what* orthopedic patients wanted from digital technologies, it was important that the technology included features that centered on the needs of each user and were interactive in terms of logging and tracking and were visually instructive (video-based) and that allowed connectivity through messaging.

#### Logging and Tracking Recovery

Interviewees perceived numerous benefits from keeping logs during the perioperative period. In the first instance, they recognized personal benefits from “logging and tracking (their) recovery” (Participant 5) to visually “see your progress” (Participant 16) and “gauge where you are and how well you’re doing” (Participant 4). This was viewed as something that would “give you the drive” to continue with the physical rehabilitation “to benefit yourself further” (Participant 9). Participants saw benefits in allowing members of the multidisciplinary team, such as surgeons and physiotherapists, to also view this information and “get an idea of when you’re starting to improve” (Participant 13). Others expanded on this, seeing shared access as an opportunity to obtain further medical expert advice on “pains or swelling...and problems with the scar like any bleeding or signs of infection” (Participant 13) and find “reassurance” related to wound healing (Participant 10). Participants reflected on the accountability that can arise from shared access to logs, both as a form of intrinsic accountability to keep “helping yourself at home...to give yourself the best chance” in the recovery period (Participant 15) and as a way of “proving that you’re doing what you’re meant to” to their surgical team (Participant 13):

Things that record what you’ve done so you can see and say “ah, I’ve achieved that, I’ve done that”...I have the incentive to go further.

Participant 11

Keeping a log of wider experiences during their perioperative journey, beyond physical activity, was also considered useful. A preoperative log of “mood, sleep deprivation and pain-management” strategies was considered important for participants to “validate your (their) mental-side” in the run-up to surgery (Participant 5). Two participants reflected on personal experiences that affected their mental health during the surgical journey, where “meditation or soothing-type app” features would have supported them “through particularly tough” preoperative pain, postoperative pain, and isolation during recovery (Participant 10). Another called for integration of an interactive “diary on the app...where you could type in how you feel...if there’s any problems” alongside “logging the pain and the level of pain” (Participant 13).

Within the logging features, patients described in detail about the user-centered information they wished “to be told by the technology” (Participant 5). Emphasis was placed on “the specific tracking...of any (form of) activity,” rather than only walking or running; being able to “compare your times or distances” (Participant 5) through “a graph or a visual” comparative feature (Participant 11); and having real-time functionality so that you can “track your progress accurately...like keeping track of your reps and weights” without relying on retrospective data entry (Participant 16). Participants also discussed share functions when it came to their logged activities where integrated competition features, such as “leader boards with friends” (Participant 12), appeared to add an incentive to engage with physiotherapy-based recovery. Combining these with “rewards and badges” (Participant 4) for logged activities appeared to reinforce patient motivation and engagement while keeping the technology user centered:

I’ll think to myself “can I do it quicker, can I go further?”...which I think are all the correct messages one needs to hear when you put it in the context of general healthy living.

Participant 11

#### I Want Something to Show Me

Video features, as an interactive method of engaging with physical activity during the surgical journey, were discussed by all participants. Preoperatively, participants reflected that videos could be used to educate patients on “exercises they’ll be expected to do” (Participant 5) and to build awareness around “the limitations, physically, that you will feel after the surgery” (Participant 8). Drawing on their experience, postoperative patients felt that being able to watch videos to practice rehabilitative exercises, preoperatively, would have given them “confidence and reassurance” to better engage with the recovery process from an earlier stage, optimizing “the entire recovery process, to give myself the best chance” (Participant 1). On the whole, participants felt video content to be of the highest value in the postoperative period, where patients can interact with instructional, surgery-specific rehabilitation advice by watching “user-friendly...video tutorials with people doing” the exercises (Participant 16). Participants discussed the integration of video-based postoperative “success stories” at various milestones of the recovery process, recognizing the power of video messages to help “visualize what you can achieve” (Participant 6) and “push me further with recovering” (Participant 16):

With each exercise there could be a video tutorial with people doing them so you can go on, click, watch the video...it could help you understand the exercise the physio(therapist) recommends...and learn how to do it properly so it’s of most benefit.

Participant 16

Along with using videos for instructional and educational purposes, participants reflected on their “changed views” (Participant 9) of integrating video call features in digital technologies for support during the perioperative period. For many, these views were linked to and influenced by the global COVID-19 pandemic. Participants described the usefulness of video calls, accepting them as a valuable and convenient form of communication while “getting used to a new normal, a different way of doing things with technology” (Participant 13). When discussing an upcoming preassessment appointment, one participant remarked that their preference for the consultation would be a video call in comparison with a proposed telephone call:

I’d be more than happy with Skype to “see them” for my appointment...I think it’s more personal, phone calls aren’t personal...I’d much prefer to Skype now instead.

Participant 13

Two postoperative patients reflected on their current experiences of undergoing video-based physiotherapy sessions in light of COVID-19 measures. These participants remarked the following:

[the content of the sessions] doesn’t differ that much from actually being in-person—you can see everything well, the resolution is good and the picture is clear, I can hear clearly.

Participant 10

everything we done the week before with the physio, we replicated on the Zoom call...everything that had been done in-person was quite easily done on the Zoom call.

Participant 14

#### Messaging Someone to Settle Your Nerves

Participants felt that the inclusion of message-based features (whether with other patients or with health care professionals) within an intervention would offer numerous benefits. Preoperative participants expressed value in communicating with other people undergoing the same surgery. This related to information-seeking needs to learn from peers, with participants discussing how they already “have looked for blogs and posts from other people going through the operation” (Participant 12) and “asked for advice...to find out what the surgery is like” (Participant 13). Coupled with searching for educational support, preoperative patients reported seeking reassurance from others before undergoing the surgery, to hear “success stories (of) people who have gone through it successfully” (Participant 17) to support their surgical decision making. This was particularly important in younger participants who wished to discuss with patients of similar ages to ask “how quick their recovery was” (Participant 15) and the level to which they retained their physical activity and functioning following surgery. One participant described their preoperative nerves without knowing “what my life is going to look like after my surgery” and considered how “having a conversation or messaging someone to settle your nerves” could help (Participant 15).

Postoperatively, participants discussed the value of sharing “experiences on a forum” (Participant 7), suggesting the integration of a patient-led “discussion area” within an app “for people who’ve gone through similar surgeries*—*whatever question it may be, they can put it on there and receive feedback from people” (Participant 16). Participants demonstrated an awareness of “mis-information or mis-interpreting the information” that may be shared (Participant 16) and acknowledged how one could become easily “disillusioned” by comparing or “judging yourself on other people’s recovery” (Participant 2). Both pre- and postoperative participants considered the morale boost that can come from communicating with peers, regardless of the stage of their surgical journey:

It’s harder when you’re on your own, but when you’re doing it alongside other people, having them to just be there as a point of reference or just to ask daft things to, that’s much easier.

Participant 5

“Messaging features” (Participant 10) could also enable two-way interactivity between the patient and a member of the multidisciplinary team, where examples discussed took various forms, from real-time “live-chat boxes” (Participant 10) to “a personal account, like Facebook messenger” (Participant 13). It was important to specify response times when it came to seeking information in this manner, with some participants desiring an “instant reply from someone” (Participant 10) for emergency purposes such as “wound healing or infection” concerns (Participant 4), whereas others considered that a “response within 24 hours...or a defined period of time” for generic questions was suitable to “fit around the (professional’s) workload” (Participant 16). Participants saw value in sharing both image- and text-based messages to aid clinical decision making, such as “how is your wound healing?” (Participant 10) or “identifying any signs of infection” (Participant 3), suggesting the value of visual connectivity in this cohort. Similar feelings of reassurance were also seen when participants discussed possible interactivity with members of the surgical multidisciplinary team:

Even the idea of (clinicians) saying “we’re here, even though it’s through technology”...it gives you a bit peace of mind.

Participant 9

### Theme 2: Directing a Descriptive and Structured Recovery Plan

Another important consideration of *what* this study wants from digital technologies was how directed, descriptive, and structured the content was. Perioperative participants expressed their desire for a digital intervention that could support them in “making the best recovery” by providing a structured and directed program with “suggestions of what you should be doing at each stage” (Participant 16). Postoperative patients discussed a “lack of direction” (Participant 16) in their current experiences following surgery, with extended periods between follow-up appointments where they lacked “the necessary, ongoing support” (Participant 2). One postoperative patient, 2 years after their total hip replacement, described gaps where “I was just winging it, really” in relation to recommended physiotherapy exercises:

there was no kind of updates with stuff when I was at home.

Participant 9

Participants reported knowing “within each stage of recovery, you should be pushing a little bit more” (Participant 15) but felt unsupported to do this. This view was especially apparent in previously physically active patients and those of a younger demographic who wanted to be challenged further to restore “functionality in the joint after surgery” (Participant 12):

all I was after was some indication of what to do to safely push on...having some indication of “this is what you need to do in this week, then move onto this”...I wanted something to show me.

Participant 4

Recommendations to provide this structured recovery program stemmed around designing “milestones...in terms of where you could expect to be after Week 1, Week 2,” with the inclusion of “physiotherapy messages” (Participant 12) and “general healthy living messages” (Participant 11). Tailoring the intervention to support a structured recovery would mean starting with “simple exercises to start the recovery and build on from there” (Participant 16). Participants described the integration of gamification features and “progression-based exercises” throughout the recovery where, over time, the program recommended “trickier exercises...working towards that final goal of being recovered” (Participant 16). Both pre- and postoperative participants viewed the capability of setting “targets and goals to work towards” as an important feature of creating a structured and directed recovery program (Participant 4). Combining goal setting with gamification features to break “(rehabilitation) down into small chunks at the start, advancing through each level” (Participant 15) and real-time messages of support such as “well done, you’ve completed this level, next it’s...” (Participant 4) were deemed motivational in giving “more people focus for what to achieve after the surgery” (Participant 5). Having a directed rehabilitation structure with set *milestones* to unlock over time also allowed participants “to feel some independence that it’s up to you to advance through the levels or reach a certain target, but with the comfort of knowing it’s still safe, you’re not pushing too hard” (Participant 12). The incorporation of safety-netting features to recover at *a safe speed* also provided reassurance for preoperative patients that they will not be pushed to “do too much too soon” (Participant 12) and compromise their outcomes following surgery.

### Theme 3: Enabling Customizable, Patient-Controlled Settings

When it came to addressing our research question of *how* patients wished to use these technologies, the benefits of having built-in, customizable, and “patient-controlled features” to enable elements of control were widely discussed (Participant 4). This ranged from wanting to “set myself my profile, choose my name...” (Participant 14) to having the ability to “build your own workout” (Participant 16) and “preference certain exercises to make it individualized to each person” (Participant 12). Interviewees perceived that customizable functionality would encourage greater engagement and a sense of accountability, meaning they better “connect with the (recovery) process” (Participant 4). One participant referenced the layout features of an app they were currently using, explaining how it was possible to “toggle the home-screen settings” to make it more personal (Participant 12):

It’s going to need a personal approach—but if you were able to toggle certain settings to make it individualized to each person, then you’ll get more successful outcomes with it and impact different people in different ways.

Participant 12

Accompanying the ability to customize aspects of physical recovery, participants also recognized benefits in preferring features relating to the mental and motivational postsurgical journey. Choosing a “more personal reminder” (Participant 7) approach to notifications was deemed constructive and supportive, with encouraging messages of “have you done your physio yet?” rather than “automated “do your physio” notifications” (Participant 12).

Having the capacity to tailor preparatory and recovery information to individual participants was widely discussed—in particular, by those who described high levels of physical activity before surgery and a wish to continue this postoperatively:

it completely depends on who you are as an individual and what you want from it (surgery)

Participant 4

Being able to “advance at a pace suitable for you” (Participant 12) during recovery was deemed imperative to restore previous “functionality of the joint” (Participant 6) and, in the process, meet individual postoperative expectations. From their experiences, some viewed rehabilitation exercises as “rather pedestrian” (Participant 6) and that “the whole process, the whole support...was geared around older and less mobile people” (Participant 4). It appeared that exercises were not designed with a younger or more active patient in mind. When it came to using technology to manage this, participants expressed desires to be able to “choose your own difficulty...to make the recovery challenging enough” (Participant 12):

(recommendations) should be determined by how active you already are...it’s no good telling me “walk 1 mile” when I’m used to walking 20! It’s the same for someone perhaps less active when they can’t functionally do it.

Participant 4

### Theme 4: Delivering General and Specific Surgical Advice in a Timely Manner

Addressing our research question around *when* digital technologies would be of most benefit, the timing (initiation point) of the intervention appeared crucial. It was discussed that technology should be initiated to meet both the pre- and postoperative information-seeking needs of participants. Specifically, preoperative interviewees wished to have explicit “sections for before surgery” (Participant 11) to seek information about the surgical procedure, to understand the best way to prepare, and to familiarize themselves with the upcoming process of recovery so as to be “already in that mindset...to the idea of the time and energy we need to invest in order to fully recover” (Participant 17). On reflection, some postoperative patients felt that their recovery would have benefited from knowing this information in advance. “Staggering the information” was also considered important, with ideas of drip feeding and building up advice in the preoperative period so that postoperatively, they would be better prepared (Participant 10):

I was ready for the off, straight away...I had it in my mind that that’s what I needed to do...you don’t want to be waiting ‘til you’re post (-operative) to hear those things.

Participant 5

Participants felt that the initiation of digital interventions should also be arranged with a sense of *generalizability* between surgical procedures so that patients undergoing any form of elective orthopedic surgery may find the preoperative information beneficial. Participants described the need for “a generic advice” hub (Participant 15) for all orthopedic patients to use, with “different tabs for different surgeries” so that patients could find surgery-specific information if they wanted (Participant 8). Two participants discussed the feasibility of having one “centralized database” (Participant 12) of exercises, breaking “the exercises down to different body parts,” and being able to easily find those that they could do to aid their recovery (Participant 16). In addition, interviewees called for holistic “general health and recovery” sections, integrating “positive health advice” that would be useful to hear throughout the perioperative process of any surgery (Participant 6). This included preoperative advice on preparation for surgery and “building muscle strength beforehand” (Participant 15), reassurance on postoperative physical rehabilitation, and “short- and long-term messages” around overall healthy living (Participant 11):

There are generic exercises that would be recommended for most joint surgeries, just to build up the muscle strength again...(and) if you had an app where you could select “hip replacement” and it provided you with “this is what exercises you should do”...it could give you more specific information.

Participant 4

## Discussion

### Principal Findings

This patient-informed study underlines the importance of obtaining orthopedic surgical patients’ perspectives in relation to the design and functionality of digital technologies to best support their recovery. By collecting both pre- and postoperative patient perspectives, we were able to clearly identify specific features and functionalities that appear to be the most desired and of most benefit in supporting this surgical cohort across the whole perioperative pathway. We addressed 3 research areas: *what* patients wanted from digital technologies, *how* they wanted to use them, and *when* their use would be of most benefit.

A consistent finding across interviews was that participants saw value in having a digital intervention to direct them through a structured plan to achieve a *successful recovery*. In terms of technology design, both prescriptive and descriptive contents were desired, where participants called for regular digital milestones to guide them and measure their journey toward recovery. Previous studies have demonstrated the benefits of continuous measurement within the recovery process following cardiac [31] and neurological surgery [32]. This feature should be considered for orthopedic interventions, where quantifying the progress can motivate patients to take active roles in their recovery [33]. Mehta et al [34] aligned this idea with reports of positive reinforcement by setting and meeting individual recovery goals following hip arthroplasty. Goal setting is a well-recognized behavior change technique that supports self-regulation skills in the change process [35,36]. In previous orthopedic studies, digital goal setting facilitated personal fulfillment and provided patients with a sense of control and accomplishment during the perioperative period [37,38]. Combining goal setting with performance feedback and the review of goals (akin to milestones within the recovery journey) has been associated with both short- and long-term intervention effectiveness [39,40]. Personalized and tailored feedback on these goals could be acknowledged as relevant and actionable, as opposed to generic advice [41]. By integrating digital strategies to help define goals within recovery, orthopedic patients may feel better supported and motivated to engage in health behavior change.

Participants valued the integration of video-based features in digital interventions, whether as a visual aid for rehabilitative exercises or to facilitate remote telemedicine consultations. Our findings support the growing popularity of video-based consultations reported in other areas of global health and social care [42-44], with participants reporting feelings of connectedness, empowerment, and reassurance through image- and video-based sharing [45-47]. The incorporation of video call features within digital health technology is gaining attention, particularly as a consequence of the global COVID-19 pandemic [43,48]. It appeared that the more prominent use of video call features, both in participants’ work and social lives, has led to greater acceptance and adoption of their use within the world of health care [48].

Another promising strategy of digital intervention design, *gamification*, has also been linked to increased user engagement [49,50]. In this study, participants’ suggestions to incorporate leaderboards and collect rewards during the postoperative recovery process echo recent findings from adult and pediatric patients undergoing orthopedic, dental, and ophthalmic surgeries [51,52] and those concerning wider eHealth design [40,53]. The use of game-like rewards and incentives has been shown to motivate and sustain health habits over time [54,55]. In wider public health initiatives, incentive-based health apps and activity-tracking programs have been associated with positive physical activity behavior change in Canada [56] and the United Kingdom [57,58]. Other successful digital health interventions have incorporated gamification features, promoting intrinsic and extrinsic motivators [59-61]. Similarities can also be recognized between these findings and wider work on persuasive systems design in relation to shaping health behaviors during the perioperative period [62-66]. Oinas-Kukkonen and Harjumaa [62] proposed that persuasion principles (including praise and rewards) should be considered as requirements in software design.

This study contributes further evidence to support gaps in the literature, which relate to the timing of intervention use, including the initiation and continuation points of intervention use. This gap has also been acknowledged in recent systematic reviews and research by Jansson et al [16], Mirkovic et al [67], and our research team [12,27]. Interventions that are initiated preoperatively and continued postoperatively were perceived as beneficial. Captivating the preoperative patient mindset and making use of the surgical teachable moment appears to be significant in encouraging perioperative behavior change and optimizing postoperative outcomes [68]. Being granted a sense of control and responsibility over their recovery by initiating and using interventions preoperatively were valued by participants. Before surgery, interviewees described the desire to customize their technology and its content to best suit their needs, thereby encouraging better engagement with the upcoming recovery process. The individualization of care pathways has been discussed in medical and surgical literature [69,70]; however, our study also highlights the importance of individualization of the *technologies* to support care delivery. Technologies that incorporated customizable features, which the patient could control and toggle according to their personal preferences, were considered another motivator for successful recovery. Participant autonomy has been shown to positively impact motivation levels and user experience, thereby improving patient care experiences [71-73]. Technology-enabled, preference-based care has improved patient and health care professional outcomes [72-74]. Technology creators may consider implementing customizable features to grant patients autonomy over aspects of their recovery [67,75].

All participants in this study discussed the impact of the global COVID-19 pandemic on the UK NHS. At the point of interview, 3 participants were undergoing technology-enabled follow-up appointments with their physiotherapist and 2 had used video call–based software to conduct their preoperative assessments with members of the surgical multidisciplinary team. Participants’ views echoed those discussed in this study on *digitally engaged patients* and recognized the multitude of ways in which technologies can be embedded within the NHS to transform surgical patient support throughout the entire perioperative journey [48]. Interactive health technologies have been credited as transformers of health care by supporting engaged self-care and promoting positive health behaviors [76]. The global pandemic has presented a unique opportunity for the creative delivery of health care. It is important that this momentum gained to adopt and use digital technologies is not lost, with the focus being continued provision of innovative surgical patient care, monitoring, and follow-up spanning the whole perioperative period [77].

### Limitations

We acknowledge that there are some limitations with this study. The intended method of in-person data collection was impacted by the COVID-19 pandemic. Although virtual call–based software enabled the replication of face-to-face interviews (ie*,* responding to verbal and nonverbal cues and building rapport) [78,79], there are some disadvantages to this interview technique that may have impacted our study. Established familiarity and participant comfort of use may have resulted in the higher number of interviews conducted over the telephone. Despite this, video calls enabled a unique snapshot into life of a patient recovering at home during the crisis and provided a fuller picture with more context than a telephone call may have done [80]. Participants currently experiencing remote consultations with members of the surgical team offered timely insights into this study. As a result of the COVID-19 pandemic, many elective orthopedic surgeries were canceled, which meant that fewer preoperative participants could be recruited and interviewed in comparison with postoperative participants. This study predominantly focused on a small sample of patients in Northern England, and as a result, the experiences shared by participants may not be representative of all care pathways across the United Kingdom. This study also focused solely on the perspectives of elective orthopedic surgical patients, and thus, the results may not be generalizable to other elective surgical specialties or acute surgeries.

### Conclusions

The results of this study have important implications for the design, functionality, application, and use of digital technologies for patients undergoing elective orthopedic surgery. By integrating digital goal-setting strategies within their recovery, patients feel better supported and motivated to engage in health behavior change to optimize surgical outcomes. The use of game-like rewards and incentives has been seen to motivate and sustain positive health habits over time. The integration of video features was acknowledged as an interactive method of engaging with physical activity during recovery and is regarded as a more personal strategy to enable follow-up consultations. This study contributes to the limited amount of existing digital health literature in this patient cohort and provides much-needed evidence relating to the optimal timing of digital interventions for elective orthopedic surgical patients. These findings should be employed in future codesign projects to enable the design and implementation of patient-focused, tailored, and targeted digital health technologies within modern health care settings.

## Appendix

Multimedia Appendix 1 Consolidated Criteria for Reporting Qualitative Research: 32-item checklist.

## Abbreviations

HRA: Health Research Authority
NHS: National Health Service
